# Mst1 and Mst2 kinases: regulations and diseases

**DOI:** 10.1186/2045-3701-3-31

**Published:** 2013-08-28

**Authors:** Funiu Qin, Jing Tian, Dawang Zhou, Lanfen Chen

**Affiliations:** 1 State Key Laboratory of Stress Cell Biology, School of Life Sciences, Xiamen University, Xiang’An South Road, Xiang’An District, Xiamen, Fujian 361102, China

**Keywords:** The Hippo pathway, Mst1, Mst2, Reactive oxygen species, YAP, Cancer, Immune diseases

## Abstract

The Hippo signaling pathway has emerged as a critical regulator for organ size control. The serine/threonine protein kinases Mst1 and Mst2, mammalian homologs of the Hippo kinase from Drosophila, play the central roles in the Hippo pathway controlling the cell proliferation, differentiation, and apoptosis during development. Mst1/2 can be activated by cellular stressors and the activation of Mst1/2 might enforce a feedback stimulation system to regulate oxidant levels through several mechanisms, in which regulation of cellular redox state might represent a tumor suppressor function of Mst1/2. As in Drosophila, murine Mst1/Mst2, in a redundant manner, negatively regulate the Yorkie ortholog YAP in multiple organs, although considerable diversification in the pathway composition and regulation is observed in some of them. Generally, loss of both Mst1 and Mst2 results in hyperproliferation and tumorigenesis that can be largely negated by the reduction or elimination of YAP. The Hippo pathway integrates with other signaling pathways e.g. Wnt and Notch pathways and coordinates with them to impact on the tumor pathogenesis and development. Furthermore, Mst1/2 kinases also act as an important regulator in immune cell activation, adhesion, migration, growth, and apoptosis. This review will focus on the recent updates on those aspects for the roles of Mst1/2 kinases.

## Introduction

The Hippo pathway plays a very important role in controlling cell proliferation and differentiation, and monitoring organ size and oncogenesis. This pathway was first discovered in *Drosophila* through genetic screens for regulators of organ size. The loss of function (LOF) mutant of the protein kinase “Hippo” exhibits tissues overgrowth and tumorigenesis, in which the increased cell number is associated with the acceleration of cell cycle progression and a failure of developmental apoptosis [1-5]. The Hippo phenotype closely resembles phenotypes of LOF mutants of the protein kinase Warts [6,7] and the small noncatlytic protein Mats [8] as well as a milder phenotype of another noncatalytic scaffold protein Salvador (Sav) [9,10]. Sav binds both Hippo and Warts, and promotes Hippo phosphorylation of Warts; Mats is another Hippo substrate that binds to and promotes Warts activation. With the activation of those downstream elements, the key role of Hippo signaling is to inhibit Yorkie [11,12], a transcriptional coactivator of proliferative and pro-survival genes. These studies in Drosophila defined a developmentally regulated growth-suppressive and proapoptotic pathway operated by the Hippo kinase. Each of the core components of this pathway is evolutionally conserved and their counterpart(s) are identified in mammalians respectively. In general, the mammalian Ste20-like kinases Mst1 and Mst2 [13,14] (Mst1/2, corresponding in Drosophila as Hippo), associated with the WW-domain scaffolding protein WW45 (corresponding in Drosophila as Sav), that binds Mst1/2 and phosphorylates Large tumor suppressor (Lats1/2, corresponding in Drosophila as Warts) [15], through their respective SARAH coiled coil domains, thereby promoting Mst1/2 phosphorylation of Lats; Mst1/2 also phosphorylates Mps one binder kinase activator-like 1 (Mob1A/B, corresponding in Drosophila as Mats) [16,17] which enhances Mob1’s ability to bind and activate Lats1/2; phospho-Mats binds to and promotes Wts/Lats autophosphorylation and activation; Lats1/2 phosphorylates Yes-associated protein (YAP, corresponding in Drosophila as Yki) [18], which promotes 14-3-3 binding to YAP, causing YAP nuclear exit, hereby inhibiting its function. Intranuclear YAP/Yki mainly promotes cell proliferation and resists cell death through the Scalloped/TEAD transcription factor(s). Loss of Mst1/Mst2 results in a YAP dependent accelerated proliferation, resistance to apoptosis and massive organ overgrowth. The details of many aspects of the Hippo signaling pathway can be found in depth discussion from several recent reviews [19-24]. In this review, we will focus on the recent updates of the roles of mammalian “Hippo” kinases, ie. Mst1 and Mst2, on the cellular redox state regulation and their involvement in organ size control, tumorigenesis, and immune regulation.

### Mst1/2 and the cellular redox state

Oxidative stress induces the activation of Mst1/2 [25]. Thioredoxin-1 (Trx1), a conserved antioxidant protein that is well known for its disulfide reductase activity, can physically associate with the SARAH domain of Mst1 in intact cells and inhibit the homodimerization and autophosphorylation of Mst1, thereby prevents Mst1 activation; whereas H2O2 abolishes this interaction and eventually causes the activation of Mst1. Thus, Trx-1 might function as a molecular switch to turn off the oxidative stress-induced activation of Mst1 [26]. Besides the Trx-1 as a redox-sensitive inhibitor of Mst1, the molecular mechanism of reactive oxygen species (ROS)-induced Mst1 activation needs to be further defined. Hippo/Mst1 kinase directly phosphorylates and activates the forkhead box proteins (FOXO), which causes expression of proapoptotic genes, such as the *FASL* and *TRAIL* genes under stress conditions. The apoptosis of cultured neurons induced by oxidative stress or by Mst1 over expression is blocked by RNAi depletion of FOXO [27]. Mst1 mediates oxidative stress-induced neuronal cell death by phosphorylating the transcription factor FOXO3 at serine 207 [27], or FOXO1 at serine 212 [28]. Mst1 and its scaffold protein Nore1 are required in cell death of granule neurons upon growth factors deprivation and neuronal activity [28]. Yuan’s group further demonstrates that oxidative stress induces the c-Abl-dependent tyrosine phosphorylation of Mst1 and increases the interaction between Mst1 and FOXO3, thereby activating the Mst1-FOXO signaling pathway, leading to cell death in both primary culture neurons and rat hippocampal neurons. These results suggest that c-Abl-Mst-FOXO signaling cascade plays an important role in cellular responses to oxidative stress and might contribute to pathological states including neurodegenerative diseases in the mammalian central nerve system (CNS) [29,30]. Indeed, Mst1 mediated FoxO3 activation in response to β-amyloid (Aβ) has been shown to mediate death of selective neuron in Alzheimer's disease (AD) [31]. Furthermore, amyotrophic lateral sclerosis (ALS) associated SOD1(G93A) mutant induces dissociation of Mat1 from a redox protein trx-1 and promotes Mst1 activation in spinal cord neurons in a reactive oxygen species-dependent manner. Genetic deficiency of Mst1 delays disease onset and extends survival in mice expressing the ALS-associated G93A mutant of human SOD1 [32].

Lim’s group recently also shows that Hippo-Foxa2 signaling pathway plays a role in peripheral lung maturation and surfactant homeostasis [33]. In the immune system, Mst1 deficient peripheral T cells have impaired FOXO1/3 and decreased FOXO protein levels indicating a crucial role of the Mst1-FOXO signaling pathway for the maintenance of naive T cell homeostasis [34]. Mst1 deficient lymphocytes and neutrophils exhibit enhanced loss of mitochondrial membrane potential and increased susceptibility to apoptosis [35]. More recently, Valis K. et al. further demonstrated that the activation of Hippo/Mst1 is able to stimulate the transcription of another proapoptotic mediator NOXA in a FOXO1-dependent Manner via acetylation of the histone proteins in the *NOXA* promoter [36]. The Hippo/Mst1-FOXO1-Noxa axis is a novel tumor suppressor pathway that controls apoptosis in cancer cells exposed to anticancer drugs such as a-TOS [36]. In contrast, a recent study demonstrates that Ras activation and mitochondrial dysfunction cooperatively stimulate production of ROS resulting in activation of JNK signaling which cooperates with oncogenic Ras to inactivate the Hippo pathway, leading to up regulation of YAP targets Unpaired (an Interleukin-6 homologue) and Wingless (a Wnt homologue) in Drosophila [37], although earlier study show activated K-Ras induces apoptosis by engaging the RASSF1A-Mst2-Lats1 pathway [38].

**Figure 1:**
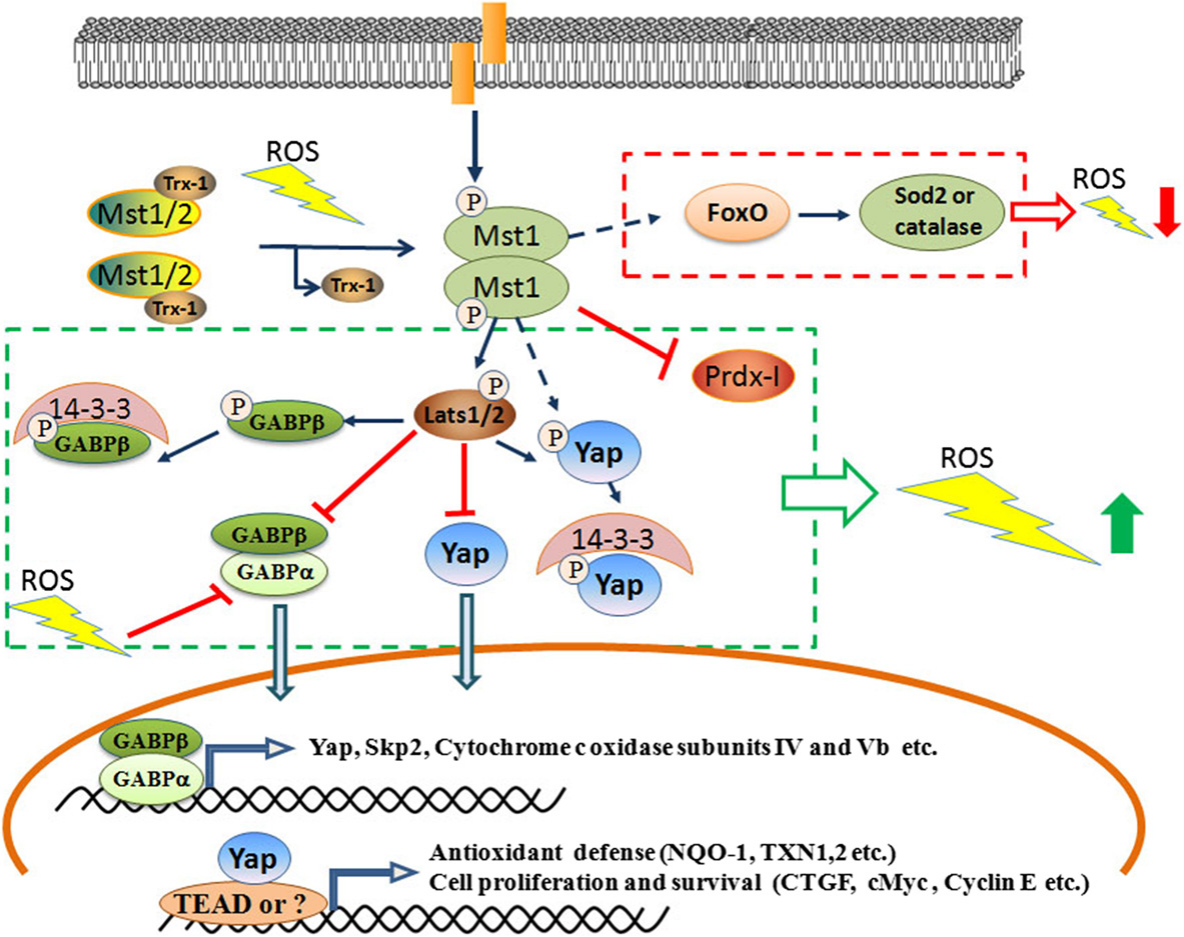
**Mammalian Mst1 and Mst2 kinases play essential role in the regulation of cellular redox state.** See text for details.

Recently, Morinaka et al. demonstrate that peroxiredoxin-1 (Prdx1), a cysteine-containing, highly conserved enzyme that reduces H2O2 to H2O and O2, interacts with Mst1 under conditions of oxidative stress and Prdx1 is required for Mst1 activation by H2O2, as knockdown of Prdx1 is associated with loss of Mst1 activity [39]. Chernoff’s group also shows that both Mst1 and Mst2 interact with Prdx1 in HEK-293 or in human hepatocarcinoma HepG2 cells under oxidative stress conditions [40]. However, the later one supports that Prdx1 represents a downstream target, rather than an upstream regulator of Mst1. Mst1 phosphorylates Prdx1 at the highly conserved Thr-183 site resulting in inactivation of Prdx1 with subsequent increased H2O2 levels in cells. As Mst1 can be activated by increased H2O2 levels, inactivation of Prdx1 resulted from the activated Mst1 might enforce a feedback stimulation system to prolong or intensify Mst1 activation. Such a feedback stimulation system, resulting in higher oxidant levels and DNA damage, might represent a tumor suppressor function of Mst1/2 to prevent the accumulation of mutations [40]. Consistently, our recent study shows that elimination of Mst1/2 from liver cells is accompanied by increased expression of a cohort of anti-oxidant enzymes important for ROS elimination [41]. The increased expression levels of those enzymes, such as glutathione reductase (GSR), NAD(P)H:quinone oxidoreductase (NQO1), γ-glutamyl-cysteine ligase (GCL, including catalytic subunit (GCLC) and modifier subunit (GCLM)), catalase (CAT), copper/zinc superoxide dismutase (SOD), cytosolic thioredoxin (Txn1) and mitochondrial thioredoxin (Txn2), promote the accumulation of glutathione (GSH). The accumulation of GSH in the Mst1/2 deficient liver results in the activation of the GA-binding protein (GABP) which is a critical transcription factor for the expression of YAP [41,42]. In addition, Mst2-Lats1 can physically bind and promotes phosphorylation of GABPβ which interrupts GABPα/β homodimerization, prevents their nuclear localization and inhibits their transcriptional activity. Thus, in addition to inhibit YAP function by phosphorylation of YAP and promoting YAP nuclear exit, Mst1/2-Lats signaling can also inhibit YAP function by downregulating its expression level [41]. In contrast to the Mst1-FOXO signaling pathway leading to the decreased ROS production, the activation of the Mst1/2 pathway inhibiting YAP in liver tissues maintains the higher levels of ROS (Figure 1). There is no doubt that oxidative stress activates Mst1/2 signaling; however the conflict effects on regulating the cellular oxidative state upon the activation of Mst1/2 are reported in different cell contexts. It is possible that the Mst-FOXO signaling pathway is predominantly activated in neuron or immune cells resulting in the decreased ROS production, whereas in other cell types, such as hepatocyte, the activation of Mst1/2-GABP-YAP signaling leads to increased ROS production. These critical but inconsistent findings indicate the importance and complexity of inter-regulation among mitochondrial function, oxidant generation and/or clearance, and the Hippo signaling pathway.

Increased production of ROS during pro-oxidant conditions would lead to Mst1/2 activation resulting in phosphorylation of GABP, inhibition of its transcription activity, and downregulation of YAP expression, consequently decreased the expression of a variety of genes that encode mitochondrial proteins and proteins with antioxidant properties, resulting in increased cellular ROS and a diminished GSH/GSSG ratio [41]. On the other hand, GABP itself helps modulate oxidative metabolism of the cell through regulating the expression of many genes necessary for cellular respiration in mitochondria, including enzymes involved in oxidative phosphorylation, such as cytochrome c oxidase subunits IV and Vb [43]. Growing evidence points that the cellular redox state and redox signaling has significant roles in regulating the metabolic fate and regenerative potential of adult tissues [44,45]. The GABP will emerge as a critical component of the Hippo signaling pathway for its role in regulating the cellular redox state and cell growth.

### The roles of Mst1/2 in organ size control and tumorigenesis

The Hippo signaling pathway is a tumor suppresser pathway. Mst1 or Mst2 single knockout mice are viable and do not exhibit obvious organ overgrowth or tumor development, whereas Mst1 and Mst2 double-knockout (DKO) mice exhibit early embryonic lethality [46,47]. To define the roles of Mst1 and Mst2 in vivo, conditional knockout mouse of Mst1 and Mst2 in variety tissues were generated and severe context-dependent phenotypes were observed (Table 1). For example, Hippo seems to control cell-cycle exit and terminal differentiation in some tissues without having major effects on organ growth, whereas in other tissues Hippo signaling maintains stem cell/progenitor compartments. The Hippo-Lats-Yorkie tumor-suppressor pathway predicated in Drosophila does not prevail in all mammalian tissues. In mammalian liver, Mst1/Mst2 negatively regulates Yap1, whereas, in mouse embryo fibroblasts (MEFs), the cell–cell contact results in Yap1 phosphorylation and nuclear exclusion equally well in wild type and Mst1/Mst2 DKO MEFs [46]; in mouse keratinocytes, Yap inactivation during cellular differentiation occurs independently of Mst1/2 and lats1/2 [48]. Thus, it appears that the wiring upstream of Yap1 and downstream of Mst1/Mst2 has been diversified considerably in mammals compared with the *Drosophila* Hippo pathway.

**Table 1:** Phenotypes of the Mst1/2 conditional knockout mice

**Affected tissue**	**Phenotypes of Mst1/2 deficient mice**	**References**
**Liver**	Dramatic hepatocyte proliferation and hepatomegaly; Development of hepatocellular carcinoma (HCC) and cholangiocarcinoma within 2 months.	46,49,50
**Intestines**	Intestinal dysplasia; An expansion of stem-like undifferentiated cells; An almost complete absence of all secretory lineages; Development of the polypoid lesions and colonic adenomas within 3 months old.	52
**Pancreas**	A significantly decrease in pancreas mass; Acinar cell atrophy; Overabundance of ductal structures; Smaller islets with abnormal α/β cell ratios in pancreas	59,60
**Heart**	Expansion of trabecular and subcompact ventricular myocardial layers; Thickened ventricular walls, and enlarged ventricular chambers without a change in myocardial cell size.	66

#### Liver

We and other groups have demonstrated that Mst1 and Mst2 are the most potent tumor suppressors in liver and a single copy of either Mst1 or Mst2 can significantly inhibit tumor formation in the liver [46,49,50]. Elimination of both alleles of Mst1 together with heterozygosity for Mst2, and vice versa, results in the development of spontaneous hepatocellular carcinomas associated with loss of the remaining wild-type Mst1 or Mst2 allele in the tumors, whereas no tumors were observed in other organs of these mice. Conditional inactivation of Mst1/Mst2 in the liver results in the immediate onset of dramatic hepatocyte proliferation and hepatomegaly followed by the development of Hepatocellular carcinoma (HCC) and cholangiocarcinoma within 2 month, in which loss of Mst1/2-dependent inhibition of YAP contributes to the liver cell proliferation and tumorigenesis.

Inactivation of Mst1/Mst2 in liver leads to the loss of YAP(Ser127) phosphorylation and increased YAP nuclear localization. Knocking-down YAP in Mst1/Mst2-deficient HCC cell lines results in massive cell death and cell cycle arrest, similarly, the restoration of Mst1 expression in these cells restores YAP(Ser127) phosphorylation and leads to cell cycle arrest and apoptosis. In contrast to *Drosophila*, Lats1/2 does not serve as the Mst1/Mst2 activated YAP kinase in hepatocytes, indicating the existence of an novel, as yet unidentified intermediary kinase downstream of Mst1/Mst2 that is critical for YAP(Ser127) phosphorylation in the liver [46]. However, our recently study shows that activation of Mst2/ Lats1 can downregulate the expression of YAP by regulating GABPβ1 phosphorylation and cytoplasmic retention in HepG2 Cells. Besides reduced YAP(Ser127) phosphorylation, the relative expression levels of YAP have also been shown significantly increased in human HCCs compared with nontumorous livers [41]. Nevertheless, both the upstream regulation of Mst1/2 and the full spectrum of Mst1/2 antiproliferative targets remain to be defined as do the relative role of these pathways in promoting hepatic carcinogenesis [51].

#### Intestines

The intestines of Mst1 or Mst2 single knockout mice are indistinguishable from their wild-type counterparts. Mst1/2 intestinal DKO mice (*Mst1*^*−/−*^*Mst2*^*fl/fl*^*-villin-Cre*) with ablation of both Mst1 and Mst2 in intestinal compartment are born normal at birth, however they develop colonic adenomas within 3 months old and can only survive for about 13 weeks (median age) accompanied by severe wasting. Both the small and large intestine of *Mst1*^*−/−*^*Mst2*^*fl/fl*^*-villin-Cre* mice exhibit an expansion of stem-like undifferentiated cells expressing high levels of CD133, Leucine-rich repeat-containing G-protein coupled receptor 5 (Lgr5) and Achaete-scute complex homolog 2 (Ascl2), which are stem cell markers in the intestine, an increased number of cells expressing CD44 and CD24, markers associated with colon cancer stem cells, and an almost complete absence of all secretory lineages. The loss of Mst1/2 in intestine decreases phosphorylation of YAP(Ser127 and Ser384) and causes an increase in both YAP abundance and nuclear localization. The hyperproliferation and loss of differentiation caused by the Mst1/2 deficiency can be entirely reversed by deleting a single YAP allele in *Mst1*^*−/−*^*Mst2*^*fl/fl*^*-villin-Cre* mouse [52]. Thus *Mst1*^*−/−*^*Mst2*^*fl/fl*^*-villin-Cre* mouse exhibits similar phenotype to the transgenic mice overexpressing YAP(Ser127Ala) in the small intestinal compartment, wherein intestinal dysplasia and loss of goblet and Paneth cells are also observed [52,53].

The inactivation of Mst1/2 in the intestine compartment to promote the hyperproliferation of intestinal stem cells and to inhibit intestinal epithelial differentiation is attributed largely to an enhancement of β-catenin action and an activation of Notch signaling. The enhanced β-catenin transcriptional activity in the intestine compartment of *Mst1*^*−/−*^*Mst2*^*fl/fl*^*-villin-Cre* mouse is evident by the increased abundance of the activated form of β-catenin (dephospho-Ser37/Thr41) and Wnt targets Lgr5 and Ascl2 [52]. The expression levels of the Notch ligand Jagged 1, mediated possibly in part through up-regulated Wnt signaling [54,55], the intranuclear Notch intracellular domain (NICD) and the abundance of Hairy and enhancer of split 1 (Hes1), a Notch target gene, are all increased in Mst1/Mst2 deficient intestine. Those evidences indicate that the Notch signaling pathway is highly activated in the intestine of *Mst1*^*−/−*^*Mst2*^*fl/fl*^*-villin-Cre* mouse. Mst1/Mst2 deficient intestines develop colonic adenomas, and unlike the polyps described in the Sav1-deficient colon [56], the polypoid lesions in the Mst1/Mst2-deficient colon do not exhibit a sawtooth/serrated architecture but hyperproliferative adenoma which might result from an activation of β-catenin and/or the inactivation of the Hippo signaling pathway in these lesions [52,57].

#### Pancreas

The Hippo pathway is necessary for proper development and to preserve homeostasis in the liver and intestines, both of which, as well as the pancreas, are developed from a primitive gut tube derived from the embryonic endoderm [58]. Thus the pancreas specific Mst1 and Mst2 conditional knockout mice using Pdx1-Cre were generated to study the effect of the Hippo pathway during mouse pancreas development. Mst1/2 pancreas-specific knockout (Mst1/2-Pdx-Cre) mice were born with no distinctive pancreatic defects at birth, however, in contrast to Mst1/2 liver-specific knockout mice with the hepatomegaly phenotype, Mst1/2-Pdx-Cre mice have a significantly decrease in pancreas mass relative to that of wild-type littermate controls at adult age [59,60]. These mice exhibit obvious morphologic alterations, including acinar cell atrophy, overabundance of ductal structures, and smaller islets with abnormal α/β cell ratios in pancreas. In brief, the pancreas became more ductal and less acinar in phenotype. Furthermore, a YAP-dependent loss of acinar cell identity and extensive disorganization in Mst1/2 deficient exocrine tissue leads to pancreatitis-like autodigestion which might result in tissue necrosis and pancreas mass decrease.

In mouse embryo, normal pancreatic differentiation is divided to two stages, the primary transition and the secondary transition. The primary transition occurring between embryonic days 9.5 and 12.5 (E9.5 and E12.5 respectively) marks the appearance of very low levels of acinar digestive enzymes and the first wave glucagon-gene and subsequently insulin-gene expressing cells. The secondary transition (between E13.5 and E16.5) characterized by intense proliferation and differentiation throughout the pancreas epithelium spans the geometric increase of acinar digestive enzymes and insulin [61]. Mst1 (but not Mst2) and YAP proteins are detected in the wild type pancreas during the secondary transition stage, and was almost undetectable at birth before returning to higher levels at postnatal day 7 (P7) and P14. Mst1/2 deficiency does not affect YAP protein levels in the embryonic pancreas, but lost of Mst1/2 was associated with higher levels of total YAP at adult age [59]. Within the adult pancreas, Yap expression is limited to the exocrine compartment, including ductal and acinar cells, whereas loss of Mst1/2 increases the YAP protein level and nuclear accumulation of nearly all exocrine cells accompanied with increased cell proliferation rate. Those evidences suggested that Mst1/2 signaling does not play a major role in pancreas organogenesis, but become functionally active during the secondary transition. The activation of Mst1/2 is required for regulating postnatal YAP levels and phosphorylation status in acinar cells to maintain differentiation [59,60].

#### Heart

It has been shown that Mst1 regulates heart size by activating its downstream kinase, Lats2, and inhibiting YAP activity, thereby attenuating compensatory cardiomyocyte growth. In cardiomyocytes, Mst1 is activated by pathological stimuli, such as hypoxia/reoxygenation in vitro and ischemia/reperfusion in vivo [62]. Mst1 mediates cardiac troponin I phosphorylation and play a critical role in the modulation of myofilament function in the heart. The function of Mst1 in cardiomyocytes can also be negatively regulated by a new identified Mst1-interacting protein protein-L-isoaspartate (D-aspartate) O-methyltransferase (PCMT1) [63]. Cardiac-specific over-expression of Mst1 in mouse results in activation of caspases, increased apoptosis and dilated cardiomyopathy, whereas the inhibition of endogenous Mst1 prevents apoptosis of cardiomyocytes and cardiac dysfunction after myocardial infarction without producing cardiac hypertrophy [62,64]. Furthermore, Del Re DP and colleagues show that Rassf1A is an endogenous activator of Mst1 in the heart and the function of Rassf1A/Mst1 pathway is different between cardiomyocytes and fibroblasts. The Rassf1A/Mst1 pathway promotes apoptosis in cardiomyocytes playing a detrimental role; while the same pathway inhibits fibroblast proliferation and cardiac hypertrophy through both cell-autonomous and autocrine/paracrine mechanisms, playing a protective role during pressure overload [65]. More recently, cardiac conditional knockout mice with either WW45, Lats2 or Mst1/2 using the Nkx2.5-cre exhibit expansion of trabecular and subcompact ventricular myocardial layers, thickened ventricular walls, and enlarged ventricular chambers without a change in myocardial cell size [66]. Yap1 protein was robustly detected in neonatal and juvenile mouse heart and declined with age. Cardiomyocyte-restricted loss of Yap1 in Fetal resulted in marked, lethal myocardial hypoplasia and decreased cardiomyocyte proliferation, whereas fetal activation of Yap1 stimulated cardiomyocyte proliferation [67]. Thus, the Mst1/2-WW45/Lats2-Yap1 pathway is critical of cardiomyocyte proliferation, cardiac morphogenesis, and myocardial trabeculation, but it does not influence physiological hypertrophic growth of cardiomyocytes during the experimental context. Gene expression profiling and chromatin immunoprecipitation revealed that Hippo signaling negatively regulates a subset of Wnt target gene in cardiomyocyte [66].

### The functions of Mst1/2 in immune system

The murine Mst1 and Mst2 kinases are most abundant in tissues of the lymphoid system. Mst1 kinase acts as an important regulator in T cell selection, adhesion, migration, growth, and apoptosis [68-73]. The Mst1 deficient mouse exhibits a reduction in white pulp, decreased numbers of total CD4^+^ T cells, CD8^+^ T cells and B220^+^ B cells and absence of marginal zone B cells. Compared to the wild type littermates, Mst1-deficient mice have much fewer CD62L^hi^/CD44^lo^ naïve peripheral T cells and a high proportion of CD62L^lo^/CD44^hi^ effector/memory T cells in tissues, such as liver and lung. Inactivation of Mst1 and Mst2 does not have obvious effect on the thymocytes development, although a lightly small size thymus is found in the *Mst1*^*−/−*^*Mst2*^*fl/fl*^*-VavCre* mouse. This might due to the very low abundance and activity of Mst1/2 kinases in double-positive (DP) cells and developmentally earlier thymocytes. Recently, patients bearing LOF mutations of Mst1 are reported with a primary immunodeficiency syndrome characterized by naïve CD4^+^and CD8^+^ T-cell lymphopenia in particular, as well as neutropenia, closely assembling with the major defect of Mst1 deficient mice in lymphocyte homeostasis. Those patients have recurrent bacterial infections, viral infections, and autoimmune manifestations with autoantibodies [35,74,75]. In contrast to defects seen with deletion of Mst1, a global deletion of Mst2 caused no changes in lymphocyte numbers in any compartment. However, the additional elimination of Mst2 in the entire hematopoietic lineage on an Mst1 deficient background (*Mst1*^*−/−*^*Mst2*^*fl/fl*^*-VavCre* mouse) causes a marked exacerbation of the deficits seen in Mst1 deficient T cells, suggesting that Mst2 might play a redundant role in lymphoid tissues during the absence of Mst1 [69]. The kinase activity of Mst1 is essence for T cell homeostasis, since the defective phenotype of Mst1/Mst2 deficiency in the lymphoid compartment can only be restored by the transgenic expression of wild type but not catalytically inactive Mst1.

Mst1-deficient naive T cells proliferate vigorously in response to TCR stimulation and have enhanced ongoing apoptosis in vivo. Mst1, but not Mst2, is greatly reduced in effector/memory T cells compared to that in naïve T cells, thus Mst1 might serves as a likely determinant of the threshold for activation of naïve T cells. Upon the T cell receptor (TCR) stimulation, the increase in tyrosine phosphorylation of CD3ζ, ZAP70, Lck, and PLCγ is similar in splenic T cells from wild-type and Mst1 deficient mice, whereas the phosphorylation of Mob1A/B observed in the wild-type T cells is lost entirely in the Mst1 deficient T cells. Elimination of Mst1 has little effect on the Lats1 carboxyl-terminal phosphorylation, Lats1/2 autophosphorylation and YAP phosphorylation in T cells. Thus the activation of Mob1A/B might serve as the effector of Mst1’s antiproliferative effect in naïve T cells [69,71]. The disruption of Mst1, or both Mst1 and Mst2, impairs the thymocyte egress and causes an accumulation of nature T cells in thymus, shown as the increased proportion of single-positive (SP) thymocytes in thymus, and a decreased number of lymphocytes in circulation. Mst1-deficient mice show defects in adhesion, homing, and intranodal migration in vivo. Furthermore, two independent pools of the ADAP/SKAP55 module, one of which associates with RAPL, Mst1, and Rap1, whereas the other interacts with RIAM, Mst1, Kindlin-3, and Talin are identified that they are independently recruited to the α- or β-chain of LFA-1 and coordinate CCR7-mediated activation of LFA-1 as well as T-cell adhesion and migration [76]. Thymocytes express multiple Rac1/2 GEFs [77], in which the deletion of Dock2 resulting in similar defects in migration, actin polarization, and Rac GTPase activation seen in the Rac1/Rac2-deficient thymocytes [78]. Mst1/Mst2 double knockout thymocytes lack the ability to activate RhoA as well as Rac, however, no evidence shows that Dock2 is a regulated downstream of Mst1/Mst2. Although the limited overlap between Dock8 and Mst1/Mst2 deficiency, loss of phospho-Mob1A/B activation of Dock8 might contribute to chemokine-stimulated Rac1 activation in Mst1/Mst2-deficient thymocytes and in turn to the failure of thymic egress [69]. More recently, Mst1 in thymocyte has also been shown to involve in LFA-1/ICAM-1-dependent high-velocity medullary migration and is required for migrating thymocytes to associate with rare populations of Aire^+^ ICAM-1^hi^ mTECs in a negatively selecting environment. Thus, Mst1 might have a key role in regulating thymocyte self-antigen scanning in the medulla [79].

## Conclusion

The mammalian Hippo pathway has generated great interests and gained significant progress in the past few years. In addition to the conserved role of growth control and tumor prevention, the Hippo pathway has also been shown to integrate with other critical signaling pathways, such as Wnt and Notch pathways and extend its function in many other critical biological events. There are still many open questions in the Hippo pathway field remained to be fully elucidated, especially the mechanism by which upstream regulators of the Hippo pathway to initiate or terminate signaling, and how the cellular redox plays a role in this process. Advances in understanding the Hippo signaling pathway regulation may not only solve the scientific questions, such as organ size control and developmental regulations, but also provide new therapeutic targets for human diseases.

## Abbreviations

DKO: Double-knockout; FoxO: Forkhead box protein; GABP: GA-binding protein; GSH: Glutathione; Lats1/2: Large tumor suppressor; LOF: Loss of function; MEFs: Mouse embryo fibroblasts; Mob1A/B: Mps one binder kinase activator-like 1; Mst1/2: Mammalian Ste20-like kinases; Prdx1: Peroxiredoxin-1; ROS: Reactive oxygen species; TCR: T cell receptor; Trx1: Thioredoxin-1; WW45: WW-domain scaffolding protein; YAP: Yes-associated protein.

## Competing interests

The authors declare that they have no competing interests.

## Authors’ contributions

All authors drafted and approved the manuscript.
